# Fitness and Physical Activity in Children and Youth with Disabilities 

**DOI:** 10.1155/2012/162648

**Published:** 2012-12-30

**Authors:** Maria A. Fragala-Pinkham, Margaret E. O'Neil, Kristie F. Bjornson, Roslyn N. Boyd

**Affiliations:** ^1^Research Center for Children with Special Health Care Needs, Franciscan Hospital for Children, Brighton, MA, USA; ^2^Department of Physical Therapy and Rehabilitation Sciences, Drexel University, Philadelphia, PA, USA; ^3^Department of Pediatrics, Seattle Children's Research Institute, University of Washington, Seattle, WA, USA; ^4^Queensland Cerebral Palsy and Rehabilitation Research Centre, The University of Queensland, Brisbane, QLD, Australia

Over the last decade, there has been a major shift in pediatric rehabilitation from an impairment-based model to activity-based interventions focusing on improving fitness, physical activity, and participation for children and youth with disabilities [1]. A catalyst for this shift is related to the emphasis on health promotion and disease prevention in the health care arena. There is growing evidence on the importance of daily physical activity for health in children and youth as well as evidence on positive health outcomes from programs that promote physical activity and fitness for children and youth; however, less information is available on these topics for children and youth with disabilities [2–6].

As rehabilitation interventions incorporate more strategies to increase fitness, physical activity, and participation in children and youth with disabilities, it is critical that outcome measures are appropriate to examine intervention effectiveness. Over the past few years, there has been an increase in measurement methodology research to ensure the feasibility, reliability, validity, and responsiveness of fitness and physical activity measures for children and youth with disabilities. It is important that pediatric rehabilitation researchers design sound intervention and measurement protocols to identify the effectiveness of activity-based interventions to improve fitness and physical activity in children and youth with disabilities. It is important that these measures are accessible and feasible for researchers and clinicians. Further, it is important that researchers articulate clear operational definitions of the fitness components (strength, endurance, flexibility, and body composition) and physical activity dimensions (frequency, duration, and intensity) that are being examined in the research. The aim of this special issue is to expand the level of knowledge about fitness interventions, physical activity participation, and measurement protocols for children and youth with disabilities.

This special issue represents an international forum of physical activity and fitness research. The articles reflect the components of the World Health Organization's International Classification of Functioning, Disability, and Health (the ICF Model) and they target a variety of disabilities and conditions of childhood including cerebral palsy, spina bifida, motor disability, and neurodevelopmental disorders. 

The ICF Model includes three personal dimensions; body structure and body function impairments, activity limitations, and participation restrictions. In this special issue, two articles focused on body structure and function impairments with one examining the potential for cardiometabolic dysfunction in youth with spina bifida and the role of physical activity and exercise while the other examined arterial structure and function in cerebral palsy. Four articles examined different aspects of activity. Two articles examined measures of physical activity; one was a systematic review of clinimetric properties of habitual physical activity measures in motor disability and one examined the feasibility of accelerometry to measure physical activity in cerebral palsy. Another article focused on functional electrical stimulation as an intervention strategy to assist in cycling activity and one examined child, family, and environmental factors that influence physical activity levels in children with special health care needs. Two articles examined participation; one in the context of play in children with cerebral palsy and one in the context of overall physical activity participation in children with neurodevelopmental disorders.

Each study illustrates the multiple aspects of physical activity and fitness that must be considered when designing an intervention program and measurement protocol to examine outcome effectiveness. Further, these papers also represent the variety of issues to consider when conducting research with children and youth with disabilities. There is a multitude of physiological, medical, functional, and environmental factors that contribute to the effectiveness and sustainability of interventions and outcomes. Also, when one conducts pediatric research, there are multiple maturational and developmental factors to consider. The articles in this special issue reflect the breadth of childhood as different researchers focused on different age ranges of childhood and adolescence.

This special issue provides a good resource to inform and facilitate future research on fitness and physical activity in children and youth with disabilities. This collection of work represents a substantial contribution to the burgeoning field of activity-based research and fitness and physical activity outcomes for children and youth with disabilities.

*Maria A. Fragala-Pinkham* *Maria A. Fragala-Pinkham* *Margaret E. O'Neil* *Margaret E. O'Neil* *Kristie F. Bjornson* *Kristie F. Bjornson* *Roslyn N. Boyd* *Roslyn N. Boyd*
